# Success factors in adaptation of newly graduated nurses: a scoping review

**DOI:** 10.1186/s12912-023-01300-1

**Published:** 2023-04-18

**Authors:** Hafidza Baharum, Aniza Ismail, Lisa McKenna, Zainah Mohamed, Roszita Ibrahim, Nor Haty Hassan

**Affiliations:** 1 Department of Community Health, Faculty of Medicine, Universiti Kebangsaan Malaysia, 56000 Kuala Lumpur, Malaysia; 2 School of Nursing and Midwifery, La Trobe University, Bundoora, Australia; 3 Department of Nursing, Faculty of Medicine, Universiti Kebangsaan Malaysia, 56000 Kuala Lumpur, Malaysia

**Keywords:** Adaptation, Scoping review, Transition, Organisation, Newly graduated nurse

## Abstract

**Background:**

Difficulties in adapting to the workplace can affect newly graduated nurses’ transition. Such nurses must adapt quickly, as it can affect their future career prospects. Therefore, this review aimed to identify the success factors that promote newly graduated nurses’ effective transition and adaptation.

**Methods:**

The Joanna Briggs Institute scoping reviews methodology was used. Data were extracted from MEDLINE, Scopus, EBSCOhost, and Web of Science publications published between 2011 and 2020. A total of 23 articles were included in this review, which comprised qualitative, quantitative, and mixed methods primary research studies focusing on the contributing factors that aided newly graduated nurses’ adaptation to the work environment during their transition period. Key emerging themes were identified with thematic analysis.

**Results:**

Three main themes were identified: (1) organisational contribution (social development, organisational culture, work characteristics, work readiness, work commitment, professional role), (2) personality traits (self-embodiment, personality masking, being proactive and confident), and (3) academic institutions (pre-entry knowledge and role of nursing faculty). Newly graduated nurses’ adaptation should begin during nursing education, be supported by the workplace organisation, and driven by the nurse’s personality. We determined that that the role of nursing education in aiding the provision of the required knowledge and actual clinical experiences to students profoundly affected developing nurses’ self-confidence levels in delivering nursing care effectively. Additionally, a warm environment supported nurses emotionally and physically.

**Conclusions:**

While organisations and educational institutions have undertaken numerous efforts to ensure that newly graduated nurses are adequately supported, the nurse’s personality and values are also equally important to ease adaptation during the transition process. Academic and workplace programs designed for newly graduated nurses should apply and emphasise this knowledge to develop and strengthen their personalities and values, especially to increase confidence and promote proactive values that facilitate newly graduated nurses’ rapid and effective adaptation to their new employment.

**Supplementary Information:**

The online version contains supplementary material available at 10.1186/s12912-023-01300-1.

## Background

Globally, issues related to new nurses remain a concern among researchers and are well-documented to ensure that nurses practise nursing safely. Such issues also affect the retention of new nurses in the workforce and profession. Often referred to as newly graduated nurses, new nurses are certified nurses who have graduated from an accredited nursing school [1]. Newly graduated nurses are also referred to as newly registered nurses (RNs) [2], newly licensed RNs (NLRNs) [3], and new graduate RNs [4]. Newly graduated nurses are generally defined as nurses with a service period of < 2 years [1, 5], although some researchers have categorised such nurses as those with < 3 years of working experience [6, 7].

The transition period for newly graduated nurses to integrate into social and professional practice within the hospital environment is challenging [8], which is largely due to it being a turning point from being nursing students to RNs [9]. During the transition period, newly graduated nurses frequently experience reality shock as they experience conflict between educational and professional values [10]. This occasionally causes their transition journey to be stressful, discouraging, and demotivating [11], leading to burnout and consequently increasing turnover [12]. Therefore, newly graduated nurses are required to adapt quickly, learn to perform their duties, adjust to their new roles and responsibilities, acquire the appropriate attitudes, fit into their work unit and organisational culture, and become accepted into the organisation to transition successfully [13].

Adaptation is related to interactions between individuals and extrinsic factors. Based on Roy’s adaptation model, adaptation is a process whereby people use conscious awareness and choice to establish integration between themselves and their environment [14]. This equilibrium between ecological and individual stimuli results in adaptive responses that contribute positively to adaptive behaviours. Gajda et al. [15] stated that adaptation is categorised into two dimensions (social and professional), while the adaptation process involves three stages. In the first stage, the newcomer familiarises and introduces themselves to the organisational culture and embraces it. The second stage encourages application of the knowledge and philosophical and technical information required to perform specific roles. Finally, the newcomer is incorporated into a work team. This third stage is considered the most challenging, as it requires individual assimilation and adherence to standards developed by group members [15].

Previous studies have reported on the adaptation process onset in the nursing context. Based on the transition stage model, transition shock for newly graduated nurses occurs within 4, 5 [16], or 6 months of service [17] based on the individual’s ability to respond to stressors. In contrast, Kramer argued that the transition–integration process occurs after 1 year of service given that the nurse is still in the learning phase in the first 6–12 months and receives full attention and support from their preceptor [18]. Nonetheless, the adaptation process is considered to have begun when the person is exposed to external or internal pressures, threats, and demands. This is because the adaptation process involves the individual’s mental responses and actions to meet needs, overcome tensions, frustrations, and conflicts successfully, and produce a harmonious relationship between their needs and living environment norms or demands [19]. However, when the adaptation period ends is less clear. Carleto et al. stated that the adaptation process spans a relatively long duration because newly graduated nurses are unable to adapt, integrate, and socialise quickly in their new organisational culture [20]. Therefore, adaptation can begin as early as the recruitment stage and can span several months to years after admission.

Organisational socialisation theory states that the adaptation phase is critical for newly graduated nurses and is a prerequisite for a successful transition process [21]. Organisations have implemented various efforts to support newly graduated nurses in passing their transitional phase smoothly. Such strategies and programs include mentoring and preceptorship [22], orientation [1, 21], nurse residency programs (NRP) [23, 24], nurse transition programs (NTP) [25], and introductory programs [26]. These initiatives are vital to prepare newly graduated nurses to face workplace environment and cultural challenges. However, intention to leave, job stress, and job satisfaction issues among newly graduated nurses remain a concern for researchers, as they significantly affect the organisation [27, 28].

Previous studies demonstrated that most transition programs span 6–12 months. In these programs, newly graduated nurses are in the ‘honeymoon’ phase and excitedly embarking on their new careers as RNs [6]. After passing the transition programs, nurses are expected to work independently in hectic work environments that involve resource constraints, high workloads, and different working cultures [29]. Subsequently, high expectations can cause transition shock among these nurses [30]. Their excitement gradually fades and new nurses frequently report feeling incompetent, unprepared, exhausted, disappointed, devalued, frustrated, and weak and powerless 5–7 months after completing their transition programs [31]. At this stage, it is crucial for nursing authorities to identify the factors required to support nurses to achieve social and professional adaptation. Late or failed adaptation responses can result in negative emotions such as fatigue, exhaustion, burnout, reality shock, early resignation or desire to resign, role conflict, and poor quality of life [32, 33]. Therefore, the transition to professional practice should be viewed from an adaptation perspective to ensure that newly graduated nurses successfully negotiate the transition period.

Newly graduated nurses should be able to integrate positively and achieve equilibrium between the multitude of encountered factors. This would aid them in becoming independent and competent although they might enter the profession from various backgrounds and with a wide range of prior experiences. However, there appears to be insufficient analysis of such nurses’ professional and social aspects of adaptation. Although one study focused on nurses’ adaptation, it examined adaptation from nursing students’ perspectives in the context of organisational socialisation and strategies to adapt to the clinical setting. That study revealed that an appropriate clinical learning environment, workplace knowledge, nursing students’ disposition, and positive encouragement from peers influenced the socialisation process and promoted smooth adaptation [34]. Other studies focused on undergraduate nursing students’ socialisation and highlighted the challenges and importance of learning how workplace relationships should be bridged [35, 36].

The research question for this review was formulated based on an identified research gap and is as follows: What are the transition success factors that might facilitate the adaptation process among newly graduated nurses? Accordingly, the review aimed to identify the transition success factors that aid newly graduated nurses in adapting professionally and socially to their workplaces. The findings can: (a) inform nursing authorities of the success factors related to adaptation that enhance success and retention, and (b) contribute to the growing body of knowledge and literature on newly graduated nurses’ adaptation and transition.

## Methods

This scoping review examined what is known about the aspects that require emphasis to facilitate newly graduated nurses’ effective adjustment professionally to specifically answer the research question. Thus, this review identified existing research on the topic and the areas of need for practice and future research aimed at increasing support for newly graduated nurses. The review protocol was guided by the Joanna Briggs Institute (JBI) scoping reviews methodology to identify knowledge gaps, map the literature, and clarify the factors associated with adaptation concepts [37, 38].

### Search strategy

The review began with a search of the relevant literature in a clinical electronic database subscribed by the National University of Malaysia digital library database service. The main keywords were derived from the research question and synonyms sourced from keywords identified in previous studies and expert suggestions [39]. The final search string was developed using Boolean operators in four major databases: Scopus, MEDLINE, EBSCOhost, and Web of Science (Additional file 1).

All included articles were screened based on the article selection criteria using the database sorting function. Studies published from January 2011 to January 2020 were included. Only English studies were included for review to avoid confusion and misunderstanding. The titles of items searched and retrieved from the database search (full text and abstract) were reviewed. Then, the relevant full-text articles were located for review. The reference lists of the articles sourced were also reviewed to locate additional relevant articles for inclusion.

### Article selection and data extraction

**Fig. 1:**
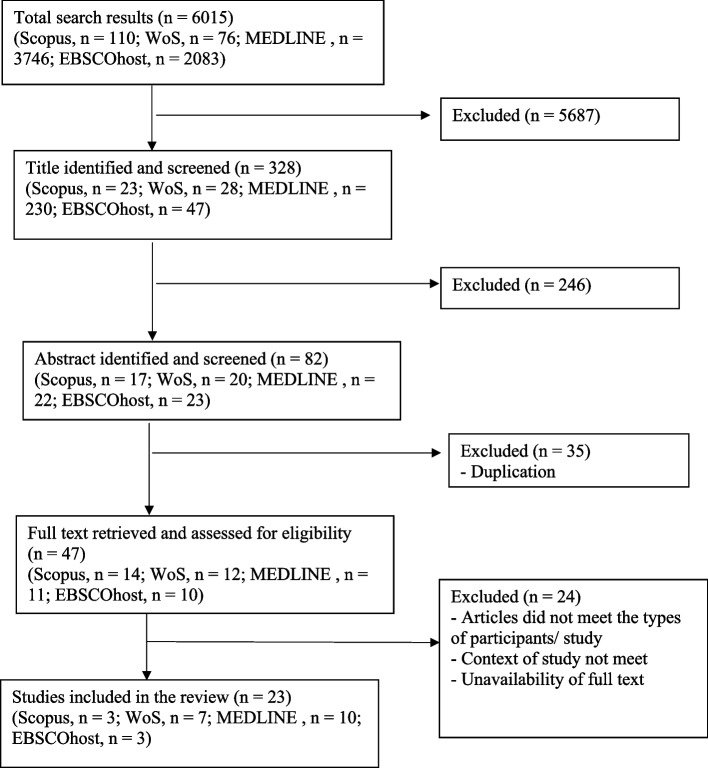
Selection and extraction of studies. Flowchart adapted from Moher D, Liberati A, Tetzlaff J, Altman DG, PRISMA Group (2009). Preferred reporting items for systematic reviews and meta-analyses: the PRISMA statement. PLoS Med 6(7): e1000097. https://doi.org/10.1371/journal.pmed1000097

Two authors performed the article selection and data extraction. Additional author reviewed the two authors’ work, and any disagreements were resolved by involving additional author. The initial search returned 6015 articles, of which 5687 articles with titles unrelated to the study scope were excluded, leaving 328 articles for abstract screening. Of the 328 articles, 246 were omitted after abstract screening, leaving 82 titles. Thirty-five of these 82 titles were removed because they were duplicates, which left 47 titles. Of these 47 articles, 24 were excluded as they did not fulfil the inclusion criteria. Hence, the full text of the 23 remaining studies were obtained and reviewed by two reviewers to ensure that all necessary information was extracted accurately (Fig. 1).

### Inclusion criteria

Twenty-three studies were included based on the study participants, phenomenon of interest, context, and type [37]. Table 1 presents the review inclusion and exclusion criteria. Studies involving newly graduated nurses working in clinical settings were included. Studies on nurses working in other settings, such as community or public health care, were excluded. All qualitative, quantitative, and mixed methods research designs examining transition factors relevant to newly graduated nurses’ adaptation were included. Literature reviews, discussion papers, and editorials were excluded.

**Table 1:** Inclusion and exclusion criteria

PICoS	Inclusion Criteria	Exclusion Criteria
Participants	Newly graduated nurses	Senior staff nurses, nursing supervisors, Preceptors, nursing students
Phenomenon of Interest	Adaptation of nurses during the transition phase	Not related to the adaptation process during the transition phase
Context	Nursing context Nurses in the clinical setting	Settings others than clinical context (nursing homes, home care, welfare)
Types of study	Qualitative studies, quantitative and mixed-methods studies	Conference abstracts, discussion papers, review papers, editorials, theses and dissertations

### Quality appraisal

While not specifically required, it is increasingly expected that studies included in scoping reviews undergo quality appraisal [40]. Thus, two evaluators rigorously validated the data extraction to confirm the accuracy of the extracted information. Both evaluators were required to agree that a study should be included, and disagreements were resolved through discussion until consensus was reached. The quality of the 23 included papers was assessed using the JBI Critical Appraisal Checklist for Systematic Reviews and Research Syntheses [37], where each article was rated as ‘yes’, ‘no’, ‘unclear’, or ‘not applicable’. No article was excluded from the review based on these assessments.

### Information collation, summarisation, and reporting

Information from the included studies was reviewed, summarised, and reported as the study findings. Codes and themes that emerged from the data were identified with inductive thematic analysis, which is a qualitative synthesis method, as the data were summarised to generate outputs in the form of themes [41]. Accordingly, the qualitative, quantitative, and mixed data were coded, translated into themes, and presented as qualitative data. The following data were extracted with Microsoft Excel: author, title, study design, objective, participants, methodology, findings, adaptation factors, and limitations. The extracted data are summarised in Table 2. Based on the data extraction, the adaptation success factors were classified into three main themes: organisational contributions, nursing academic institutions, and personality characteristics.

**Table 2:** Included articles exploring adaptation of newly graduated nurses

Author (year, country)	Objectives	Study designs and Methods	Findings	Transition factors for adaptation	Limitations
Abiodun, et al. [42] South Africa	To investigate use of an Instant Messaging application (WhatsApp) community of practice to support graduate nurses in their first year of practice in the Western Cape, South Africa	Quantitative approach; Employing self-developed questionnaire based on the Technology Acceptance Model Participants 198 final year Bachelor of Nursing students and 150 employed nurse graduates	Factors supporting graduate nurses in their first year of practice: 1. Interactions with alumni, 2. Bridging and bonding social capital, 3. Professional integration 4. Sense of belonging 5. Application of theory to practice	Social-emotional support Social construct Social belonging Pre-entry knowledge/experience	Numerous confounders may exist such as different levels of support in community service placement sites which the researchers were not able to quantify during the study
Ashton [1] United States	To explore adaptation of new registered nurses using the Roy adaptation model as the guiding conceptual framework	A quantitative approach using cross-sectional design employing single item adjustment scale, the occupational fatigue exhaustion recovery (OFER) scale, and the job-related affective well-being Scale (JAWS) Participants 250 registered nurses in North Carolina (NC)	Being in a formal orientation period significantly supported newly graduated nurses overall adaptation Factors facilitating the adaptation: 1. Personal attributes 2. Characteristic of work setting 3. Social support 4. Nursing education	Self-Confidence Socio-emotional support Shift hours Long working hours Pre entry- knowledge/experience	Single item adjustment scale has low validity and reliability to measure the adaptation. These measurements did not comprehensively capture nor adequately measure new RNs’ adaptation
Bisholt [22] Sweden	To describe and analyse how recently graduated nurses are socialised into the profession	Qualitative approach using ethnography through participant observations and interviews Participants 16 novice nurses working at a county hospital in the central part of Sweden	Physicians doubted nursing knowledge and occupational performance of newly graduated nurses because they were unable to prove their professional abilities Stress between nursing academia and organisation. In nursing education, the ideology of nursing was prominent, but within the profession emphasis was on good occupational skills Hierarchy culture- newly graduated nurses felt being excluded in the team. Seniors did not acknowledge their existence, ignore and blame newly graduated nurses for the mistake. Confidence level deteriorated	Nursing faculty role Pre-entry knowledge/experience Masking personality self-confidence	The study focused on an investigation of a small number of new graduated nurses
Chandler [43] United States	To focused on the new graduates’ perspective of the processes that enabled them to successfully integrate into their new role: to learn the processes necessary for a successful transition to describe effective supports for newly graduated nurses to develop the knowledge, skills, and attitudes needed to progress through the first year of practice	A qualitative descriptive design Participants 36 nurses who had graduated from associate degree (20%) and baccalaureate (80%) programs	Three themes were identified: ‘‘They were there for me,’’ ‘‘There are no stupid questions,’’ ‘‘Nurturing the seeds.’’	Social emotional support Social construct Constructive feedback Welcoming culture Team work Work delegation Prioritising Time management Nursing faculty role knowledge/experience- newly graduated nurses perceived well prepared	There was no explicit explanation for the difference in adaptation processes between nurses with an associate degree and those in baccalaureate programs
Feng and Tsai [44] China	To explore socialisation experiences of new graduate baccalaureate nurses to practising nurses	Qualitative descriptive approach Data collected using semi-structured, open-ended, in-depth interviews analysed by content analysis Participants Seven newly graduated nurses working in four medical centers in Taiwan	Practicum inadequately exposed newly graduated nurses to actual responsibilities and clinical experiences. Newly graduated nurses perceived organisational socialisation process involved interpersonal relationships and adapting themselves with ward rules and culture was the hardest work Socialisation process involved three themes: ‘Learning by doing’ was the strategy that helped newly graduated nurses’ transition from ‘overwhelming chaos’ to ‘being an insider’	Social construct Social acceptance Coping skill (learning by doing) Proactive Confidence	This study involved small sample; thus, the results cannot be generalised
Frögéli et al. [45] Sweden	To prospectively investigate how socialisation processes relates to experiences of stress among newly graduated nurses during the first three months of professional working life	Quantitative approach using longitudinal study with employing Stress and Energy Questionnaire), Gener al Questionnaire for Psychological and Social Factors at Work), Needs Satisfaction and Frustration Scale Participants 264 newly graduated Swedish nurses who started their first jobs during the period of the study	Socialisation processes affected newly graduated nurses’ experiences of stress. Factors to reduce stress to improve socialisation process involved: Role clarity Task mastery skills Social acceptance Proactive. Onboarding phase was different between individuals. Newly graduated nurses who were in late phase of onboarding scored high in task mastery and role clarity but lower in social acceptance	Social acceptance Role clarity Task management Proactive	Causality of effects cannot be determined
Hunter and Cook [46] New Zealand	To explore new graduate nurses’ experiences of professional socialisation by registered nurses in hospital-based practice settings, and identify strategies that support professional identity development	A qualitative descriptive design using semi-structured interviews Participants 5 newly registered nurses employed within one New Zealand region for less than six months were recruited	Three themes describe the nurses' experiences of professional socialisation: ‘Lessons from the wilderness' ‘Life in the wild’ ‘Belonging to a wolf pack’	Social support working atmosphere Feedback Self-confidence	Small sample size and homogenous participants in aspect of gender, age and ethnicity
Kramer, et al. [47] United States	To identify to what extent Nurse Residency Programs reflected the professional socialisation To determine which components, strategies, and activities of Nurses Residency Program were most effective in Newly Licensed Registered Nurses socialisation into professional practice	A mixed-method design Descriptive, quantitative data obtained from 37- items Residency Program Questionnaires (RPQs). Small group or individual interviews of newly graduated nurses was performed Participants Descriptive, quantitative data obtained from 34 hospitals Qualitative data obtained from 330 newly graduated nurses, 401 preceptors, 138 managers, and 38 educators	Components in the Nurse Residency Programs (NRPs) that reflected the professional socialisation process: Precepted role (to provide the situated learning, positive feedback/motivation to restore confidence, and guide to solve problems, task mastery, delegation and work prioritisation)- effective strategies for newly graduated nurses’ integration Reflective seminars, Skill acquisition, Reflective practice sessions, Evidence-based management projects, Clinical coaching– mentoring sessions	Social-emotional support Received constructive feedback Work performance Self-confidence	Small group or individual interviewed session is time consuming, energy and resources Challenging in scheduling a group (discussion goes beyond the schedule time)
Kramer et al., [14] United States	To identify effective components and strategies of Nurse Residency Programs (NRP) in each area	Qualitative approach using individual and focus group interview Participants 907 nurses in 20 Magnet hospitals with NRPs operative for at least 3 years participated in individual or small group interviews and 82 participant observations	Factors to promote integration in the transition phase were: Provide nursing students with experience of professional practice role and management of clinical situations Support them with discussion and activities to help them prioritise and delegate tasks, Conflict resolution—improve social development 4. Constructive feedback can restore and augment self-confidence	Prioritising skills Decision-making Problem-solving Communication Collaborative relationship Feedback Work delegation Get work done Confidence values	Small group or individual interviewed session is time consuming, energy and resources Challenging in scheduling a group (discussion goes beyond the schedule time)
Lalonde et al. [48] Canada	To explore relationships between preceptor characteristics (emotional intelligence (EI), personality (P) and cognitive intelligence (IQ)) and new graduate nurse socialisation outcomes regarding turnover intent, job satisfaction, role conflict and ambiguity	A quantitative approach using cross-sectional design employing demographic questions, Nursing Emotional Intelligence Scale (NEIS), Cattell Culture Fair Intelligence Test and International Personality Item Pool (IPIP) short scale to measure preceptors EI, IQ and personality, respectively Participants 41 preceptors and 44 new graduates completed a quantitative survey at the end of their preceptorship program	Preceptor personality traits of openness, conscientiousness and emotional stability were significantly related to new graduate nurses who reported greater turnover intent, job dissatisfaction, role conflict and ambiguity No significant relationships were noted between preceptor EI and IQ and the outcome of new graduate nurses	Role clarity Social-emotional support	Eligible nurses may not have been sampled or participated because there were changes in newly graduated nurse’s recruitment practices in Ontario during the study period
Lee et al. [49] China	To obtain a comprehensive understanding of the transition process of newly graduated nurses in Taiwan	Qualitative phenomenological approach using focus group interviews Participants 16 novice nurses from one teaching hospital in the central part of Taiwan	Concentrating to become insiders was important to survive and adapt during the transition phase. This phenomenon was characterised by four themes identified for being accepted: ‘being new as being weak’, ‘masking myself’, ‘internalising the unreasonable’ and ‘transforming myself to get a position’	Social acceptance Masking personality Self-embodiment	Generalisation is limited as it only involves sample in teaching hospitals
Leong et al. [50] Singapore	To explore perceptions of newly graduated nurses of their experiences of role transition. To examine implications for managers in terms of employee training, development and retention	Qualitative constructivist grounded theory approach using semi- structured interviews Participants 26 nursing students across five hospitals	Two major constructs appear to play an important part in the transition process: Learning how to fit in (facilitated by social interaction including observing and questioning others and seeking out social support) Aligning personal with professional and organisational identities (displaying positive attitudes, developing professional identity)	Socio-emotional support Social construct Working atmosphere Masking personality Self-confidence	The study investigated the graduated nurses and preceptors’ experiences and did not examine the senior hospital managers, human resource managers and nursing educators who may have contributed additional understanding to the data
Li et al. [51] China	To investigate newly graduated Chinese nurses' intention to leave their jobs and to explore association of intention to leave with nurse characteristics, per- son– environment fit, and social support	Quantitative approach using cross-sectional design employing single item of negative events, six- item Turnover Intention Scale, Perceived Person Environment Fit Scale, 10-item Proactive Personality Scale, and Multidimensional Scale of Perceived Social Support (MSPSS), Participants 1313 newly graduated nurses from 18 hospitals in six provinces	Reasons of newly graduated Chinese nurses considered leaving their jobs: Low degree of person- organisation fit High level of education Exposed to negative workplace Proactive personality	Social-emotional support Working atmosphere- deteriorated Self-confidence Shift hours Proactive	Cross sectional design did not permit causality measurements. Single question to measure participants' experiences of negative events could not reveal the exact relationship between these variables and NGNs' intention to leave
Malouf and West [52] Australia	To provide insight into how Australian New Graduate Nurses (NGNs) experienced their transition to acute care nursing practice	Qualitative approach using serial in-depth interviews Participants Nine intensive interviews were conducted among newly graduated nurses who employed in public and private hospital	Needs of newly graduate nurses in adaptation process in the context of socialisation: Establish relationships to build up the sense of being insider Should not to appear ‘stupid' in front of staff members Reduce the ward rotation -to reduce the stress of fitting-in for the beginner	Social belonging Working atmosphere	There was no explicit explanation for the difference in adaptation processes between nurses employed in public and private hospital
Ohr et al. [53] Australia	To investigate if current onboarding process influenced organisational socialisation of new graduate nurses and midwives into the workforce	Quantitative approach using cross-sectional design employing 41 items on researcher developed questionnaires Participants 170 novice nurses who commenced their transition program in the health District in January and February 2017	Onboarding practices in the context of organisational socialisation were: Relationship building was the main key in organisational socialisation Ability to learn workplace cultural norms Social support from preceptor However: Study address the inconsistency in the structure and content of orientation programs Current onboarding did not adequately provide strategies to build relationships for new graduates within their work environments-preceptor have role ambiguity	Social-emotional support Welcoming culture Working atmosphere Prioritising skills Coping skills Time management Problem solving Communication skills Team work	The study focused on an investigation of a small number of new graduated nurses
Pettersson, and Glasdam [54] Sweden	To explore newly employed nurses’ socialisation in the process of introduction into an oncological clinic from the perspectives of unit managers and newly employed nurses	Qualitative approach using semi-structured interviews Participants Written introductory material and interviews with seven newly graduated nurses and two-unit managers	Newly graduated nurses were socialised gradually through mirroring their supervisors in their role as nurse. Patients were function as an object for training purposes. Patients also being as the communication object for newly graduated nurses socialised with all staff	Social-emotional support Social construct Working atmosphere	There is difference between what people are doing in situ and what they say about their practices. Narratives change over time and it is articulate in space and time. However, this study does not capture these differences; neither does it capture the perspectives of supervisors
Qiao et al [55] China	To examine relationships between demographic characteristics, sources of nursing stress and coping strategies, and psychological well-being within graduate nurses	Quantitative approach using four questionnaires; Demographic Questionnaire, The Nursing Stress Scale (NSS; [56], The Brief Cope Questionnaire [57], and the General Health Questionnaire (12-version, GHQ- 12 [58];) Participants 96 participants from four hospitals in central China	Organisational sources of stress: Dealing with death and dying was the most common workplace stressor, Heavy workload, Feeling a lack of adequate preparation Adaptive coping strategies to handle work stress were: Planning Acceptance Positive reframing	Work allocation Work characteristic Social acceptance Self-confidence Socio-emotional support	Some instruments used were developed in Western countries, and they may not have been validated for use with a mainland Chinese population
Rush et al. [59] Canada	To examine relationships between selected components of new graduate nurse transition programs and transition experiences	Quantitative approach employing online survey using The Casey Fink Graduate Nurse Experience Survey Participants 1008 new registered nurses working in acute care	Orientation and transition program component predictors of new graduate workplace integration: Orientation length (should be at least four weeks in length) Average number of hours worked in a two- week period (communication and leadership skills of newly graduated nurses who work at least 49 h in a two-week period significantly higher as compared to newly graduated nurses working 48 h and less) Percentage of preceptor shifts (statistically insignificant)	Socio-emotional support Long working hour Working atmosphere Prioritising skills Communication skills	Low response rate of 26% may have resulted in a biased sample Many unexplained variances in transition scores
Sparacino, [7] United States	To identify nursing faculty behaviors that reduced stress and anxiety experienced by new graduate nurses as they transcended from role of student to professional registered nurse	Qualitative grounded theory approach using telephone interviews Participants 13 new registered nurses who successfully passed the National Council Licensure Examination—Registered Nurse (NCLEX-RN) on the first attempt	Faculty behaviours that helped prepare nursing students meet organisational demands and facilitate adaptation process during transition: Caring (newly graduated nurses ease of seeking help from faculty) Rigor (newly graduated nurses valued the nursing faculty who adhered to strict policies, assignments, appearance and punctuality), Experience (use of simulations with critical cases derived from real experience taught students how to use critical thinking and reasoning skills in high stress situations), Knowledge (vast knowledge, real-life case studies, use of multiple technological venues in the classroom and clinical setting, motivated students to study harder), Professionalism (know how to act and behave professionally- help in social development)	Pre-entry knowledge Nursing faculty role Self-confidence Proactive	Study limitations include the researcher’s previously held assumptions, the participant pool, and use of grounded theory Supplementary research is needed to further validate the theory
Tastan et al. [60] Turkey	To identify factors affecting the transition period of newly graduated nurses	Quantitative approach using survey Participants 263 newly graduated nurses working in a military education and research hospital	To facilitate newly graduated nurses’ adaptations to their new roles and in improving their learning: 1. Being/participating in the orientation programs (engagement with the preceptor) newly graduated nurses experienced reality shock in their working situations: Inadequate preparation in nursing program for their future professional lives Lack of support- working with nurses who were unwilling to help Longer working hours (more than 12 h) negatively affected their performance and work- life balance	Social acceptance Social support Long working hours Pre-entry knowledge/experience	No validity and reliability tests in the Turkish scale Long data collection may cause measurement and time period bias
Tomietto et al. [61] Italy	To determine which organisational socialisation contents affect turnover intention in newcomer nurses within their first 2 years of employment	Quantitative approach with cross- sectional design employing organisational socialisation inventory (OSI), and four items turnover intention scale by Kelloway (1999) Participants 156 newly graduated nurses	Identified important aspects for newly graduated nurses to focus during the onboarding process to help adaptation with organisational demands: Need to understand written or unwritten rules and culture to regulate organisational life in the first 6 months of employment Need to focus on relationships with co- workers Focus on individual level of task mastery in the 7–12-month period Focus on professional development opportunities	Nurse Faculty role Social construct Work performance- Task mastery Role clarity	This study employed a cross-sectional design, while organisational socialisation needs to be explored as a longitudinal phenomenon
Wahab et al. [20] Singapore	To explore new graduate nurses' accounts of resilience and facilitating and impeding factors in building their resilience	Descriptive qualitative design using photovoice and focus group interview Participants 9 new graduate nurses who completed degrees from a Singapore university	Factors impeded development of newly graduated nurses’ resilience to facilitate the adaptation process: Resilience was persevering and overcoming the obstacles Resilience was accepting one’s responsibilities and fulfilling them Resilience was adapting to new situations Resilience was taking control of own learning Factors facilitating adaptation process during the transition phase: 1. Participating in orientation program: engaging/working with preceptor, constructive feedback helped with more resilience and gaining confidence 2 Participating in the residency program: effective communication and conflict management to prepare newly graduated nurses to survive in the clinical area Warm and culture supported adjustment process	Working atmosphere Prioritising skills Time management Problem solving skills Communication skills	Recruitment of participants only from nursing graduates from only one university and one hospital in Singapore Small sample size
Yildiz and Ergün [62] Turkey	To reveal transition experiences of nurses in the first year of their profession	Qualitative approach using semi-Structured, in- depth individual interviews Participants 30 newly graduated nurses, working in three Training and Research Hospitals, two University Hospitals and three Private Hospitals	Experiences of nurses in their first professional year: Emotional (experienced frustration and intense anxiety), Sociocultural and developmental (development of professional identity, being accepted by the team, balancing work and private life and transferring education received into practice), Physical and intellectual (transferring knowledge they learned during their education to practice) Newly graduated nurses easily adapted in the transition phase if they: Received support, constructive feedback, sincerity and consistency of relationships Received appropriate expectations from seniors off newly graduated nurses Had the ability to implement and transfer knowledge learned in nursing education to practice Had the ability to achieve person-organisation fit	Social acceptance Social emotional support Social construct Pre-entry knowledge/experience Work allocation Feedback	Results cannot be generalised because study only reflects the opinions of newly graduated nurses who participated in the study

## Findings

### Background of the included articles

Twenty-three studies were included in the review. Five studies were conducted in the US, four in China, three in Sweden, and two studies each were conducted in Australia, Canada, Singapore, and Turkey. One study each was conducted in Italy, New Zealand, and South Africa. There were 10 quantitative studies, 12 qualitative studies, and one mixed methods study (Table 2).

### Workplace organisational commitment

#### Social development

The main drivers of newly graduated nurses’ workplace adaptation were socio-emotional support, social construction, social acceptance, and sense of belonging. Social support is an important aspect in creating a sense of relaxation and security and increasing morale and belonging, which facilitated newly graduated nurses’ adaptation [2, 23, 42, 54, 59]. Workplace organisations should be encouraged to provide information and instrumental support to enhance newly graduated nurses’ abilities to develop coping skills. Providing support will enable nurses to build effective relationships with supervisors and colleagues, handle time/priority management, reduce stress and anxiety, increase self-confidence, and develop the ability to absorb workplace cultural norms [29, 30].

The social construct includes bridging and bonding of connectivity between newly graduated nurses with their supervisors and co-workers. Newly graduated nurses’ interactions with their social community facilitated professional integration and promoted togetherness values, which created unity among co-workers, provided a sense of security, support, and belonging, and encourage affection [42, 54, 61]. Unfortunately, the expectation of being accepted as a team member was often challenging for newly graduated nurses if they were unable to interact and communicate effectively with their colleagues.

Newly graduated nurses believed that learning how to bridge the gap between knowing and practising was easier than learning how to behave appropriately and deal with people in the workplace [55, 60]. The nurses encountered difficulties in finding their place within groups and being friendly with senior nurses due to a gap in relationships between new and senior nurses [44, 49]. This situation rendered it difficult for newly graduated nurses to learn and understand their job requirements. In turn, the newly graduated nurses were unable to meet their workplace demands.

Greetings and personal experience sharing were micro-interactions that promoted social bonding and comfort newly graduated nurses as they felt accepted by the community [42, 63]. Furthermore, newly graduated nurses felt accepted by their team when they gained staff recognition and were praised for their occupational achievements [26]. Colleagues’ acceptance indicates newcomers’ positive socialisation into the organisation, which facilitates the acquisition of high-quality knowledge and relationships [45]. When newcomers feel welcomed and respected, they feel accepted into the team and are able to survive in hectic work environments. Positive relationships with social communities are pivotal to newly graduated nurses’ self-confidence and work enjoyment [64].

Newly graduated nurses emphasised the importance of becoming integrated, feeling like a part of a social community, and developing a sense of belonging in environments that initially felt intimidating [44, 52]. Problem- or idea-sharing and opportunities for building relationships with other nurses create a sense of belonging [42]. New nurses acknowledged that being in a group established a supportive environment, increased their self-esteem and self-confidence, and promoted a sense of ownership [44, 49]. Therefore, positive relationship development and support systems ensured pleasant and less stressful work environments and were associated with positive adaptation. Conclusively, sufficient support, positive social construction, acceptance as an insider, and a feeling of belonging among newly graduated nurses aids in the onboarding process and is reflected in high retention rates.

#### Organisational culture

Organisational culture and social capital are inter-related. Social and cultural continuity is achieved via socialisation [65]. In the newly graduated nurses’ adaptation context, the reviewed studies mainly discussed organisational socialisation, new employees’ learning process when adapting to work culture, work policies and rules, and the knowledge, skills, and behaviours required to transition from outsider to insider [66]. Newly graduated nurses were required to possess the ability to learn workplace cultural norms as a part of the adjustment process [53].

A welcoming environment is crucial to make newly graduated nurses feel welcomed and appreciated and aid them in making the right start. A friendly and welcoming atmosphere is a part of daily micro-relations that aid the alleviation of feelings of isolation [42]. A welcoming atmosphere facilitates incorporation into the organisational culture from day 1. Newly graduated nurses should be facilitated to meet other staff, as this is essential to build relationships, develop convenient workspaces, participate in organisational activities, and create welcoming environments. A positive and supportive atmosphere is important in clinical practice to reduce stress and adapt to organisational culture [24, 42, 50–53].

A warm ward culture also supports newly graduated nurses’ adjustment to new environments and aids the alleviation of challenging workloads, addressing learning needs, and increasing confidence [24]. Newly graduated nurses opined that professional staff’s willingness to address any type of question without prejudice positively affected their confidence levels and social interactions between them and other professional staff [43]. Nevertheless, especially in Asian cultures, it was reported that senior nurses used their power unreasonably by constantly scolding, embarrassing, criticising, and laughing at newly graduated nurses who asked questions or made mistakes in their work [49]. Furthermore, frequent ward rotations can also exert high stress on newly graduated nurses, as they need to establish adaptation within teams and environments regularly [52, 55]. Therefore, it is recommended that senior nurses be professional, provide supportive, constructive feedback, and give newly graduated nurses opportunities and time to learn and become familiar with work norms and co-worker behaviours.

#### Workplace characteristics

Newly graduated nurses’ supervisors should assign tasks wisely and provide learning support during the transition phase. Assigning complex tasks to newly graduated nurses undergoing transition could affect their abilities to make critical and accurate decisions, which could potentially endanger patient safety [59, 62]. Newly graduated nurses reported that dealing with death and dying patients was the most common workplace stressor, as they were not psychologically prepared to deal with dying patients [55]. Such situations can weaken confidence and increase stress, anxiety, and depression levels [55, 64]. Furthermore, newly graduated nurses experienced physical disorders and often felt nauseated if they were assigned excessive tasks and responsibilities during their transition [64]. The organisational socialisation model emphasises that appropriate workload allocation is a significant component in the adjustment phase. Appropriate allocation can enable newly graduated nurses to effectively and safely manage their tasks [21]. Therefore, newly graduated nurses undergoing the transition process should not be allocated tasks involving care for patients who require complicated treatment.

Working hours and shift and work rotations can influence newly graduated nurses’ perceived adjustment tolerance and can cause chronic and acute occupational fatigue. New graduates who worked longer hours, for example, > 49 h in 2 weeks, experienced smoother transitions [2]. Smoother transition occurs as new graduates are more engaged in the culture of practice and have opportunities to expand their experience. Contrastingly, other studies reported that long working hours affected newly graduated nurses’ quality of life and performance levels. Statistically, long working hours were significantly associated with lower job satisfaction and increased intention to leave among newly graduated nurses [60].

Newly graduated nurses who worked long hours encountered difficulties in integrating and transitioning to work [59]. Nurses who worked > 10 h per day experienced fatigue, sleep deprivation, and potentially had more needlestick injuries [63]. Compared with nurses who worked regular working hours, nurses who worked night shifts were affected differently in the context of person–environment fit, job satisfaction, and health status [2, 51]. One study reported that low job satisfaction, poor sleep quality, chronic fatigue, psychological problems, and cardiovascular symptoms were prominent among newly graduated nurses who worked night shifts [51]. Therefore, nurses engaged in night shift rotations require special attention, such as shortened or flexible working hours or reduced workloads, to improve their health, experience satisfaction, and increase their desire to work at night.

#### Work readiness

Newly graduated nurses acknowledged that job readiness determined their successful workplace transition and integration. Coping skills, organising, prioritising, time management, decision-making, problem-solving, communication skills, collaborative relationships, teamwork integration, feedback, motivation, and work delegation skills were categorised as necessary for job readiness and required in the onboarding process [53]. Chandler et al. [43] argued that nursing schools should emphasise these essential job readiness skills so that they could be practised in the workplace.

Feng [44] examined coping strategies from the adaptive behaviour perspective. Newly graduated nurses with excellent coping strategies had professional role clarity, increased self-confidence, and reduced job stress during the transition phase [34]. Good coping strategies aided new graduate nurses in building resilience and developing the ability to manage challenging circumstances when evolving from an outsider to an insider [24, 67].

Newly graduated nurses must prioritise and organise skills to evaluate their responsibilities to complete assignments with priority before developing professional nursing performance. It is essential for newly graduated nurses to organise and prioritise tasks related to time management skills to adapt to an unpredictable workplace [68]. By learning task prioritisation and wise time management, nurses can keep track of their responsibilities and reduce tension [24]. Nonetheless, both supervisors and newly graduated nurses reported difficulty in simultaneously prioritising and organising [23]. Moreover, newly graduated nurses frequently struggled during the transition phase, as they lacked conflict resolution skills [23].

Accompanying transition based on respectful feedback, effective socialisation improves confidence and skills in managing disputes [62]. The preceptor must provide feedback and guidance via positive and constructive feedback to foster a conducive learning environment [27]. Preceptors and newly graduated nurses frequently cited feedback workshops and the NRP Generation Pact as effective strategies to receive positive feedback [23]. The Generation Pact feedback system is an agreement between newly graduated nurses and supervisors to provide daily or frequent feedback to each other on the performance of various aspects of professional nursing [27]. This strategy encourages newly graduated nurses to identify means of obtaining the feedback required to maintain or restore their self-confidence.

Problem-solving requires confidence and decision-making skills [18]. Conflict resolution, effective decision-making, and coping skills are inter-related like a chain in problem-solving. Newly graduated nurses encounter problem-solving challenges as they have difficulty determining their patients’ conditions [18]. Therefore, a community of practice group would present a support system that can provide advice to newly graduated nurses [42]. The problem-solving ability drives an individual to think objectively and critically and aids them in making wise decisions. Thus, newly graduated nurses can manage their stress levels.

Communication skills are crucial in the transition and onboarding processes [24, 59]. Good communication skills aid newly graduated nurses during task delegation, as performing the job requires clear communication and trust [69]. A newly graduated nurse who lacks the expertise and knowledge to delegate appropriate care risks jeopardising the patient and potentially losing their licence to practise [18]. Therefore, task delegation is a fundamental competency and basic skill for newly graduated nurses. Task delegation is related to constructive resolution determined by critical thinking and decision-making skills [18]. Deciding on the appropriate action aids newly graduated nurses in managing their stress levels and potentially promotes adaptation.

Newly graduated nurses perceived that effective communication skills were essential in the transition–integration process to establish collaborative nurse–physician relationships [18]. Good communication also aids newly graduated nurses’ socialisation, especially in building social networks and facilitating teamwork integration [27]. An interdisciplinary patient care cycle such as SBAR (Situations, Backgrounds, Assessments, and Recommendations), communication, and telephone call techniques were suggested as effective collaboration enhancement strategies [18]. These activities enable new graduates to improve communication, practice leadership, support network expansion, and gain professional satisfaction [59].

The data analysis demonstrated that all sub-themes in the work readiness component were interconnected. The main component influencing professional adaptation was effective communication. Hence, it can be concluded that communication skills are fundamental to a successful organisation and a requirement for effective adaptation.

### Professional role

The key aspects of the onboarding process are work performance, practice development, role clarity, and task mastery involved in professional roles. Knowledge acquisition encompasses finding, gathering, and refining information for practice development and is the main component of work performance [70]. Newly graduated nurses can learn from their peers’ experiences. Competency can be enhanced by advice and information shared by seniors or supervisors. Actively seeking information and involvement with other staff members can yield better outcomes to increase knowledge, professionalism, and work performance [42].

Newly graduated nurses can accomplish their tasks effectively when they have job clarity and understand the required standards, responsibilities, guidelines, and organised work protocols [46, 61]. Role clarity among newly graduated nurses is associated with excellent work performance, high job satisfaction, and low turnover rate [45, 48]. Newly graduated nurses must understand their job scope, roles, and responsibilities to optimise their work performance. Enhancing work performance aids in maintaining job satisfaction and long-term retention [23, 61]. Nurses should not be a ‘Jack-of-all-trades’ when adjusting to new workplaces. Newly graduated nurses might lack practical experience and skills at the beginning stage; therefore, it is not appropriate for them to perform multiple tasks in the adaptation phase. Once newly graduated nurses understand their roles and develop expertise in their roles, they can master the basic skills required in the nursing profession [45]. Mastering their role demonstrates newly graduated nurses’ commitment to their profession and organisation [61].

### Nursing education contribution

#### Pre-entry knowledge/experience

The knowledge and skills acquired in nursing school can help newly graduated nurses focus on adding new skills over time [43]. Newly graduated nurses’ abilities to relate and implement their nursing education knowledge and clinical experiences to be applied in employment practice significantly aid the improvement of their work and are key to successfully facilitating the adaptation process during transition [7, 42, 62]. However, newly graduated nurses did not feel well-prepared by their nursing education, as they were unable to integrate their nursing education knowledge with clinical practice. This prompted feelings of a lack of knowledge and clinical experience [2, 26, 44, 60, 62]. One study reported differences between nursing education professional values and workplace organisational values [44]. Nursing ideology (nursing theory, nursing diagnoses, evidence-based care, development of critical thinking) is prominent in nursing education, but in practice, good occupational skills are emphasised (practical/technical, complete tasks on time) [26, 44]. Newly graduated nurses reported that the key factors hindering adjustment during transition were the feeling that they lacked knowledge and clinical experience and simultaneously facing overwhelming chaos (high workload, staff shortage) [44] while being required to complete given tasks within an allotted time. This constraint caused newly graduated nurses to feel disorganised, helpless, exhausted, and frustrated and in turn undermined their self-confidence as professional nurses [44].

Therefore, nursing programs should include topics related to students’ preparation for their future professional lives [60]. Furthermore, emphasising work readiness-related educational topics and providing students with experience with professional practice roles and managing clinical situations at the beginning of their transition period were recommended to promote integration in the transition phase [54]. Students could be assigned actual responsibilities in providing nursing care services with instructor support to aid their prioritisation of multiple tasks and task delegation to other workers [27]. Newly hired nurses who were introduced to their work environment during their preparation stages were more confident and accustomed to the new professional world.

#### Nursing faculty’s role

Nursing faculty are important in facilitating newly graduated nurses’ adaptation when transiting from being students to RNs. The Faculty Attributes with Confidence, Equilibrium, and Success (FACES) Theory was developed to contextualise faculty behaviours and responsibilities [7]. FACES encourages nursing authorities to educate students adequately for professional practice. Nursing schools should promote being knowledgeable, professional, caring, and strict teachers, having rigorous rules and protocols, and outstanding teaching plans to ensure that newly graduated nurses adapt quickly during their transition. Newly graduated nurses appreciated the role of nursing education that adhered to rigorous rules and policies to teach them self-discipline, demonstrated caring values by aiding the application of their knowledge, and provided knowledge and experiences on nursing practices to bolster their confidence and ability to find and learn new information independently. Thus, professional faculty behaviours aid newly graduated nurses in aligning themselves in working environments and building and adapting to healthcare industry social structures [7].

### Newly graduated nurses’ personality values

Attitude, personality, behaviour, and manner of thinking are expressed through human decisions when determining positive or negative actions [71]. Newly graduated nurses with positive attitudes and personalities can manage themselves efficiently. Such nurses are psychologically prepared to face stressful situations and successfully make adjustments [26, 55]. New employees can accelerate their adaptation process by recognising behaviours that help them fulfil expectations, learn organisational principles and values, and gain social approval [66], which is recognised as person–organisation fit [52].

### Proactive personality

Newly graduated nurses should be diligent in seeking information to effectively reduce misconceptions about their new employment and work organisation. Curiosity motivates newly graduated nurses to actively seek knowledge and understand the organisational culture and standards [72]. The desire to learn and perform responsibilities proactively is a coping strategy that determines how quickly newly graduated nurses adapt to workplace demands [44]. Empirical evidence proved that a proactive personality aids newly graduated nurses in developing resilience, increasing self-confidence, and coping with stressful situations, and reduces turnover [44, 45]. As mentioned earlier, feelings of a lack of knowledge and clinical experience weaken newly graduated nurses’ self-esteem and self-confidence. To increase competence, newly graduated nurses can learn through self-direction [44], which is difficult, as learning new knowledge and skills requires much time and enduring hardships [43, 44]. Nevertheless, new employees’ proactive attributes are critical to discipline, increasing self-confidence, and assisting with the handling of stressful circumstances when adjusting to a new career.

In contrast, other researchers reported that a proactive personality did not affect newly graduated nurses’ decisions to leave their profession when exposed to hostile work environments and not achieving person–environment fit [51]. Nurses with higher proactivity attributes will likely be more willing to make changes when encountering unpleasant situations. Organisational culture influenced newly graduated nurses’ adaptation more than proactive values [51]. Nevertheless, it is believed that a proactive personality is essential when adjusting favourably to an organisation and community [73]. Likewise, a proactive personality facilitates a nurse’s adaptation to becoming an insider.

### Confidence value

The concept of self-confidence is an individual’s trust in their own ability to achieve objectives and perform tasks and activities efficiently and effectively [74]. Newly graduated nurses are required to possess self-confidence to develop competency, overcome issues, and be resilient against individual and external stimuli [24, 44]. However, newly graduated nurses confronted confidence crises due to a fear of harming patients and a lack of confidence in taking responsibility, which arose from a lack of knowledge and clinical skills. Reality generates stress, such as senior nurses’ expectations and perceived chaos at work, which results in significantly decreased self-confidence and self-esteem among newly graduated nurses [4]. This condition can cause emotional fluctuations as new nurses confront stress, boredom, feelings of isolation, and occasional physical and psychological weakness, which in turn can cause early burnout [62].

Preceptorship programs are a strategy used to increase and restore newly graduated nurses’ self-confidence, where preceptors provide constant support and development, guide problem-solving, create supportive learning environments, provide constructive feedback, and provide consistent support regarding clinical skills and knowledge [23, 46]. Additionally, the role of nursing education in providing knowledge and exposing nursing students to clinical practice can aid newly graduated nurses in developing and increasing their confidence as professional nurses. These personality values are particularly important to aid adjustment and survival during transition. Newly graduated nurses with adequate self-confidence can communicate and engage with other healthcare team members, which effectively leads to professional partnerships [24]. Additionally, self-confidence aids newly graduated nurses in delegating tasks appropriately, making correct decisions, and prioritising and coordinating their daily activities [27]. Therefore, self-confidence and adaptive behaviour are inter-related.

### Self-embodiment and self-awareness

Self-embodiment and self-awareness are key strategies used by newly graduate nurses to develop and fit into social groups, thus facilitating adaptation to complex clinical environments and ward cultures [25]. Newly graduated nurses must demonstrate their ability to master nursing practices and generate positive impressions to their seniors [61]. Furthermore, newly graduated nurses are encouraged to seize opportunities to collaborate with other staff to facilitate learning of the work group culture [23]. Such attitudes aid newly graduated nurses in becoming self-aware of their surrounding environment, work culture, and colleagues’ reactions and feedback to maximise the probability of acceptance into the social group and receiving support when needed [26].

### Personality masking

Social hierarchy culture is still practised in Asian countries and is perceived as the norm, whereas it can be considered horizontal violence in non-Asian cultures. Unpredictable leadership behaviours and negative workplace cultures prevent newly graduated nurses from learning how to adapt by aligning personal, professional, and organisational identities [46]. Newly graduated nurses in Asia, reported that they were often scolded for no rational reason, risked being ‘yelled at in front of others’ if they failed to adapt to ward norms, and that senior nurses were searching for aspects to criticise and publicly blame when tasks were not performed well or mistakes were made [44, 50]. This situation can worsen newly graduated nurses’ confidence and thus they may suffer from vulnerability and incompetence. Such behaviour can be a challenge for newly graduated nurses to adapt to and be accepted as insiders.

‘Covering themselves’ is a strategy newly graduated nurses use to be accepted as members of an organisation and can protect them in difficult and adverse situations. Newly graduated nurses reportedly covered themselves by hiding their natural personalities, concealing feelings and facial expressions, interacting carefully, and refraining from synchronising interpersonal relationships with senior nurses to harmonise relationships [49]. Newly graduated nurses were often advised not to display anger even if scolded and that putting on a ‘fake smile’ and displaying positive attitudes could attract supervisor praise and encouragement [50]. Nurses are considered more resilient by taking control of their own learning and situation management [24]. Newly graduated nurses with a strong sense of identity and ability are good at managing feelings and balancing emotions and are thus better able to recover from negative attitudes and unprofessionalism [25]. Eliminating unhealthy cultures in an organisational structure by proposing culture-specific and consistent preceptorship education is encouraged in organisations. Furthermore, treating newly graduated nurses as insiders may aid the creation of a positive work environment and aid smooth adaptation [49].

## Discussion

**Fig. 2:**
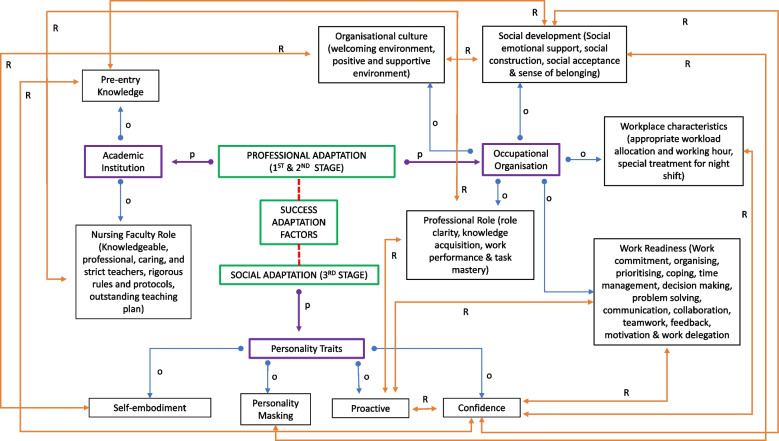
Adaptation success factors model

The main purpose of this study was to review the factors of successful transition related to newly graduated nurses’ adaptation. These factors were observed from the perspective of adaptation introduced by Gajda et al. [15]. The nursing education institution is involved in the first stage of adaptation, when newly graduated nurses are required to familiarise themselves and interact with and embrace organisational cultures, rules, and protocols. Workplace organisations are involved in the second stage of adaptation, where newly graduated nurses need philosophical and technical information to work as professional nurses. Both education and workplace institutions contribute to professional adaptation. The third stage of adaptation is perceived as more challenging, as newly graduated nurses are required to assimilate to the standards developed by a group and incorporate those standards into team work. At this stage, newly graduated nurses’ personality traits are key in facilitating their socialisation process. The data analysis revealed that the findings could be linked and summarised in an adaptation success factor model (Fig. 2).

Nursing education institution and workplace organisational efforts to provide professional adaptation for newly graduated nurses are inter-related. However, undergraduate nursing programs may not adequately prepare nursing students to be practice-ready upon course completion [75]. Some newly graduated nurses experienced difficulty in applying the theories learned in their education institutions to clinical situations and received insufficient experience and opportunities to perform in clinical practice [26, 76, 77]. Thus, they encountered difficulty in integrating what they had learnt in the education institution with their work practice. A training style learning method, rather than classroom style, may aid students and newly graduated nurses in linking their learned knowledge directly to the practical skills needed for the job [77].

Pre-entry knowledge and clinical experience often influence the adaptation process. Nursing students are first exposed to the clinical environment by their nursing education institutions, which provide them with practical experience. Newly graduated nurses perceived that nursing students might need more access to clinical learning environments [7], such as clinical simulations or emergency scenarios, during their study period to gain additional clinical experience and exposure [78]. The clinical environment encompasses simulations and practical tasks that provide learners with learning experiences and knowledge. Knowledge aids newly graduated nurses in feeling confident and contributes to self-learning. The clinical environment presents unparalleled opportunities to develop theoretical knowledge [79] and practical skills, specifically aiding the decision-making and critical thinking skills [34] professional nurses need. Newly graduated nurses can draw on experience to aid their adjustment to new work environments, as they would have a clearer understanding of their work demands and concerns [80].

The knowledge and skills acquired from nursing school and occupational organisation (social development, work readiness, workplace characteristics) are the main driving force behind newly graduated nurses’ confident personalities. Nurses’ self-confidence develops in two phases. First, they acquire theoretical knowledge and critical thinking to support appropriate decision-making. In the second phase, they integrate their evidence-based learning in the workplace and education institution to develop feelings of being insiders and create a sense of workplace belonging [81]. Academic institutions should aid newly graduated nurses in building their confidence, impart clinical knowledge and experience, and bridge the gap between clinical theory and practice. Nursing education must provide knowledge and insights into actual workplace contexts to build confidence and prepare students as clinicians [82]. The workplace organisation should strengthen newly graduated nurses’ confidence and thus increase their commitment and job readiness, assist in professional role development and social coaching, and encourage newly graduated nurses to adapt to their work culture [28].

Appropriate work allocation is linked to confidence level. Newly graduated nurses will feel stressed if they are assigned high-risk tasks, such as dealing with chronically ill patients or patients who are on the verge of dying. Such tasks generate feelings of sadness, worry, frustration, helplessness, and guilt, and can even gradually decrease a nurse’s confidence [83]. Newly graduated nurses commonly experience feelings of anxiety, dissatisfaction, low self-esteem, and a lack of confidence [84]. The perception of being unprepared and lacking clinical expertise can trigger these negative effects [85]. In the transition phase, newly graduated nurses are considered to possess the expected work efficiency if they acquire professional skills in their work readiness, as this was a factor in achieving positive work results and aiding feelings of greater confidence, which subsequently aided the adaptation process [86].

Chesser-Smyth and Long [74] reported that newly graduated nurses’ self-confidence decreased during nursing education, especially in the clinical practicum course. This situation consequently discouraged independent action and led to newly graduated nurses’ heavy reliance on senior staff. Furthermore, a lack of self-confidence magnifies the fear of communicating with other disciplines, leading to the inability to prioritise tasks [3]. Simultaneously, such nurses struggle to delegate tasks and attempt to delay or avoid conversations [87]. Based on these findings, it is asserted that academic institutions are important for preparing students with the requisite skills and clinical expertise for their final year before they join the nursing profession.

Familiarity with workplace-related phenomena, knowledge enhancement through questioning and self-exploration, clinical skills learnt from personal or others’ experiences, validation (feedback from other experienced nurses, physicians, and managers), taking responsibility to increase capability, mutual interaction with patients and the work environment, and personal creativity are efforts and strategies for newly graduated nurses to increase confidence [88]. These strategies are closely related to a proactive attitude, where being proactive can enhance feelings of self-worth and inspire confidence in newly graduated nurses, invite more positive attitudes, and thus eliminate job stress [89]. A proactive personality eases the nurse’s adaptation to becoming an insider. This aids newly graduated nurses in actively seeking knowledge and understanding organisational culture and standards, developing resilience, and coping with stressful situations, and reduces turnover rates [45, 72, 73]. As the proactive value is closely related to hardiness, the characteristics of this trait are not specific. New employees’ proactive attributes are critical for handling stressful circumstances when adjusting to a new career, suggesting that proactiveness and hardiness build on each other in a virtuous cycle and create a strong sense of energy and effort, which thus facilitates the adaptation process.

Workplace organisation programs should be implemented at educational institutions, and include feedback workshops and the NRP Generation Pact, which are effective strategies for receiving positive feedback [3]. Praise or positive feedback are associated with increased motivation and confidence and are a factor in employees moving towards their goals, ensuring that they remain on the right track and work hard to continue their success. Therefore, nurses who are confident that they can cope with their new job demands are more capable of dealing with job stress [90] and tend to increase their professional adjustment levels.

Flexibility skills, which include self-embodiment and personality maksing, are associated with social construction and feelings of acceptance. Both newly graduated nurses and supervisors should consider building trust, communicating respectfully, practising honesty, creating team spirit, and being approachable and sociable [91]. In Asian countries, newly graduated nurses can easily feel alienated in a group, powerless, and perceive that being new is weak [49]. This situation presents the opportunity for senior nurses to bully and threaten new nurses verbally or physically. Newly graduated nurses without power and a voice cannot resist the violence, yet must continue to practise independently. Therefore, this scenario forces newly graduated nurses to adapt to their environments, compelling them to strive to achieve their goals and be accepted by the team. Contrastingly, Western countries consider workplace incivility, bullying, and workplace violence to be horizontal violence, which is a key contributing factor in decreased job satisfaction, increased stress, high turnover rates, and negative effects on newly graduated nurses [92–94]. Therefore, the organisation must provide and create a positive and healthy functional working environment to support newly graduated nurses in their adaptation process. Newly graduated nurses need social support, guidance, and recognition as they encounter difficulties in social construction and internalisation when becoming insiders [2, 30].

## Conclusion

A nurse’s transition from new graduate to professional must be considered an adaptation that allows individuals to successfully negotiate the process. Generally, adaptation must be supported and emphasised at the education institution level, empowered and strengthened in the organisational workplace, and boosted by a positive personality to effectively aid new nurses in achieving self-adjustment. Therefore, effective or successful transition factors should be viewed in the broader context, which includes academic institution contribution, workplace organisation contribution, and newly graduated nurses’ personalities.

Although various efforts have been made to improve new nurses’ performance and aid the transition process, newly graduated nurses’ personalities and soft skills are obvious key elements that aid in ensuring that every effort by both institutions is successful in the adjustment process. High self-confidence and proactivity render newly graduated nurses more resilient and able to adjust to demanding work environments. This review encourages educational institutions to aid nurses in developing self-assurance and proactive attitudes by providing extensive knowledge, experience, and insights into the real working world. Workplace organisations should prioritise the improvement of newly graduated nurses’ organisational skills, enhancement of their commitment and willingness to work, assistance with professional role development, and social guidance. Emphasising these elements can indirectly improve new nurses’ positive characteristics. Therefore, educational institution and work organisation partnerships are encouraged to create a continuum of adaptation improvement, particularly to foster and strengthen newly graduated nurses’ positive personalities in facilitating the adaptation process during transition. However, there remains a paucity of research specifically examining personality and soft skill involvement in enhancing the adaptation process.

Furthermore, newly graduated nurses’ adaptation processes may differ depending on their specialty. However, the studies chosen for this review did not specifically mention such distinctions. In the future, it is recommended that adaptation across units or departments be examined. The study scope provided insights into the success factors that facilitate transition, specifically to aid newly graduated nurses in achieving self-adjustment effectively. Thus, studies with stronger designs are recommended to consolidate these practices and determine the elements that should be included.

### Limitations

The search was limited to university-subscribed databases. Hence, potentially relevant search keywords and databases might have been overlooked. Additionally, only English language studies were included, which excluded related studies in other languages.

## Supplementary Information

**Additional file 1.**

## Data Availability

All data generated or analysed during this study are included in this article.
