# Supplementary material for: Success factors in adaptation of newly graduated nurses: a scoping review
Source: BMC Nurs. 2023 Apr 18;22:125. doi: 10.1186/s12912-023-01300-1 (PMC10111715; doi:10.1186/s12912-023-01300-1)
Supplement: Supplementary file 1 — Additional file 1. [file 12912_2023_1300_MOESM1_ESM.docx]

Additional file 1: The review search string

| **Database** | **Search string** |
| --- | --- |
| Ovid MEDLINE | ("adapt*" or "adjust*" or "fit" or "fit-in" or "social*" or "integrate*" or "acclimate*" or "accommodate*" or "harmony*" or "familiar*").mp. [mp=title, abstract, full text, caption text] ("transit*" OR "progress*" OR "evolution" OR "convert*" OR "passage" OR "transform*" OR "alter*") *").mp. [mp=title, abstract, full text, caption text] AND ("new* nurse*" or "new* employ* nurse*" or "new* register* nurse*" or "new* graduate* nurse*") |
| WOS | TI= ("adapt*" OR "adjust*" OR "fit" OR "fit-in" OR "social*" OR "integrate*" OR "acclimate*" OR "accommodate*" OR "harmony*" OR "familiar*" ) AND ( "transit*" OR "progress*" OR "evolution" OR "convert*" OR "passage" OR "transform*" OR "alter*" ) AND ( "new* nurse*" OR "new* employ* nurse*" OR "new* register* nurse*" OR "new* graduate* nurse*") |
| Scopus | TITLE-ABS-KEY ( ( "adapt*" OR "adjust*" OR "fit" OR "fit-in" OR "social*" OR "integrate*" OR "acclimate*" OR "accommodate*" OR "harmony*" OR "familiar*" ) AND ( "transit*" OR "progress*" OR "evolution" OR "convert*" OR "passage" OR "transform*" OR "alter*" ) AND ( "new* nurse*" OR "new* employ* nurse*" OR "new* register* nurse*" OR "new* graduate* nurse*" ) ) |
| EBSCOHOST | ( "adapt*" OR "adjust*" OR "fit" OR "fit-in" OR "social*" OR "integrate*" OR "acclimate*" OR "accommodate*" OR "harmony*" OR "familiar*" ) AND ( ("transit*" OR "progress*" OR "evolution" OR "convert*" OR "passage" OR "transform*" OR "alter*" ) AND ( "new* nurse*" OR "new* employ* nurse*" OR "new* register* nurse*" OR "new* graduate* nurse*") |
